# Last Resort Antibiotics Costs and Reimbursement Analysis of Real-Life ICU Patients with Pneumonia Caused by Multidrug-Resistant Gram-Negative Bacteria in Germany

**DOI:** 10.3390/healthcare10122546

**Published:** 2022-12-15

**Authors:** Julia Jeck, Sebastian M. Wingen-Heimann, Florian Jakobs, Jennifer Franz, Christoph T. Baltin, Anna Kron, Boris Böll, Matthias Kochanek, Oliver A. Cornely, Florian Kron

**Affiliations:** 1 VITIS Healthcare Group, Am Morsdorfer Hof 12, 50933 Cologne, Germany; 2 Department I of Internal Medicine, Faculty of Medicine and University Hospital Cologne, University of Cologne, Kerpener Straße 62, 50937 Cologne, Germany; 3 KCM KompetenzCentrum für Medizinoekonomie, FOM University of Applied Sciences, Herkulesstraße 32, 45127 Essen, Germany; 4 Department of Haematology and Stem Cell Transplantation, Faculty of Medicine and Essen University Hospital, University of Duisburg-Essen, Hufelandstraße 55, 45147 Essen, Germany; 5 Center for Integrated Oncology (CIO ABCD), Faculty of Medicine and University Hospital Cologne, University of Cologne, Kerpener Straße 62, 50937 Cologne, Germany; 6 Department of Orthopedics and Trauma Surgery, Faculty of Medicine and University Hospital Cologne, University of Cologne, Kerpener Straße 62, 50937 Cologne, Germany; 7 National Network Genomic Medicine Lung Cancer, University Hospital Cologne, Kerpener Straße 62, 50937 Cologne, Germany; 8 Clinical Trials Centre Cologne (ZKS Köln), Faculty of Medicine and University Hospital Cologne, University of Cologne, Gleueler Straße 269, 50935 Cologne, Germany; 9 Translational Research, Cologne Excellence Cluster on Cellular Stress Responses in Aging-Associated Diseases (CECAD), Faculty of Medicine and University Hospital Cologne, University of Cologne, Joseph-Stelzmann-Straße 26, 50931 Cologne, Germany; 10 Excellence Center for Medical Mycology (ECMM), Faculty of Medicine and University Hospital Cologne, University of Cologne, Kerpener Straße 62, 50937 Cologne, Germany

**Keywords:** last resort antibiotics, reimbursement, multidrug-resistant Gram-negative bacteria, MDR-GNB

## Abstract

Multidrug-resistant Gram-negative bacteria (MDR-GNB) cause serious infections and aggravate disease progression. Last resort antibiotics are effective against MDR-GNB and are reimbursed by flat rates based on German diagnosis-related groups (G-DRG). From a hospital management perspective, this analysis compared hospital reimbursement for last resort antibiotics with their acquisition costs to outline potential funding gaps. Retrospective analyses based on medical charts and real-life reimbursement data included patients with pneumonia due to MDR-GNB treated in intensive care units (ICU) of a German tertiary care hospital (University Hospital Cologne) between January 2017 and December 2020. Drug-associated hospital reimbursement of G-DRG was compared with drug acquisition costs based on preliminarily approved last resort antibiotics (cefiderocol, ceftazidime-avibactam, ceftolozane-tazobactam, and imipenem-cilastatin-relebactam) according to label. Funding gaps were determined for the treatment of *Enterobacterales*, *Pseudomonas aeruginosa*, *Acinetobacter baumannii*, and mixed infections, respectively. Most of the 31 patients were infected with *Enterobacterales* (*n* = 15; 48.4%) and *P. aeruginosa* (*n* = 13; 41.9%). Drug-associated G-DRG reimbursement varied from 44.50 EUR (mixed infection of *P. aeruginosa* and *Enterobacterales*) to 2265.27 EUR (*P. aeruginosa*; mixed infection of *P. aeruginosa* and *Enterobacterales*). Drug acquisition costs ranged from 3284.40 EUR in ceftazidime-avibactam (minimum duration) to 15,827.01 EUR for imipenem-cilastatin-relebactam (maximum duration). Underfunding was found for all MDR-GNB, reaching from 1019.13 EUR (*P. aeruginosa*; mixed infection of *P. aeruginosa* and *Enterobacterales*) to 14,591.24 EUR (*Enterobacterales*). This analysis revealed the underfunding of last resort antibiotics in German hospital treatment. Insufficient reimbursement implies less research in this field, leading to a more frequent use of inappropriate antibiotics. The cycle closes as this contributes to the development of multi-drug resistant bacteria.

## 1. Background

Pneumonia caused by multidrug-resistant Gram-negative bacteria (MDR-GNB) is associated with both high morbidity and high mortality rates. This is especially relevant for patients in ICU. The treatment of these multi-morbid inpatients most often requires a prolonged length of stay and increased treatment costs [1]. In pneumonia, highest mortality rates were demonstrated in a sub-cohort of hospital-acquired pneumonia (HAP) patients requiring mechanical ventilation (vHAP) [2]. Isolated pathogens causing ventilator-associated pneumonia (VAP) were even associated with a 5–10% higher resistance rate to drugs than pathogens found in HAP [3].

Recent analyses reported *Escherichia coli*, *Klebsiella pneumoniae*, *Pseudomonas aeruginosa*, *Stenotrophomonas maltophilia*, and *Acinetobacter baumannii* to be the most common species of MDR-GNB [4,5,6]. Predisposing factors of MDR-GNB pneumonia associated with critically ill patients include, e.g., immunosuppressive therapy, haemodialysis, prior infection or colonisation with MDR-GNB, antibiotic therapy, and hospitalisation for more than two days within the prior 90 days [7,8]. The use of carbapenems, broad-spectrum cephalosporins, and fluoroquinolones further increases the risk of developing MDR *P. aeruginosa* pneumonia [9]. Detected MDR-GNB indicates the usage of last resort antibiotics as they may still be effective when resistances against common antibiotics exist [10,11] Timely and appropriate therapy are well-known factors improving outcome, ideally facilitated by rapid diagnostics identifying resistance genes [12].

ICU treatment is a major cost-driver in the inpatient setting [13]. Evidence of costs and reimbursements in ICU patients with MDR pneumonia, however, is limited. In Germany, inpatient antimicrobial treatment is currently reimbursed by flat rates incorporated in diagnosis-related groups (DRG). The aim of our study was analysing the funding of last resort antibiotics in ICU patients with pneumonia due to MDR-GNB in a German hospital by comparing drug acquisition costs and DRG reimbursement.

## 2. Methods

Taking a hospital management perspective, real-life German DRG (G-DRG) tariff data of ICU patients with pneumonia due to MDR-GNB were analysed. According to the Robert Koch-Institute (RKI), ‘multidrug-resistant’ bacteria are pathogens with resistances to at least three out of four antibiotic groups including acylureidopenicillins, third and fourth generation cephalosporins, carbapenems, and fluoroquinolones [14]. Due to preliminary categorisation by the Federal Joint Committee, four last resort antibiotics were included in this analysis: Cefiderocol, ceftazidime-avibactam, ceftolozane-tazobactam, and imipenem-cilastatin-relebactam [15,16,17,18]. Cases were not differentiated by HAP/VAP/vHAP, as they do not affect the grouping in the G-DRG system, and thus do not influence reimbursement.

### 2.1. Hospital Reimbursement Analyses

The relevant patient cohort was determined by analysing pseudonymised medical chart data retrieved from the data warehouse of the University Hospital Cologne. Patients being discharged in the 4-year period between January 2017 and December 2020 were included and systematically evaluated. Further inclusion criteria were predetermined: patients ≥ 18 years, combination of ICD-10 codes J15 (pneumonia by bacteria, not classified elsewhere) and U81 (Gram-negative pathogens with certain antibiotic resistances that require special therapeutic or hygienic measures), and the operation and procedure (OPS) code 8-98f (elaborate intensive medical complex treatment (basic procedure)).

Medical chart data were categorised according to critical-priority pathogens registered in the World Health Organisation Pathogen Priority List which is also supported by the RKI: *Enterobacterales*, *P. aeruginosa*, and *A. baumannii* [10,19]. Additional categories were built for patients with mixed infections of MDR-GNB. Most frequent G-DRG codes of the respective categories were exemplarily evaluated to achieve most representative results. Cost categories 4b, pre-calculated by the German Institute for Remuneration in Hospitals (InEK), were focussed to identify drug-associated hospital reimbursement [20]. The case mix was evaluated to gain further insights on the severity and resource utilisation of hospital cases.

### 2.2. Acquisition Cost Analyses

Identified drug-associated reimbursement of most frequent G-DRG tariffs was opposed to acquisition costs of last resort antibiotics per MDR-GNB category. Acquisition costs were quantified according to Lauer Taxe, which is the German reference list for pharmaceutical price information. This allowed to identify and quantify potential funding gaps in the treatment with last resort antibiotics. Product labels of last resort antibiotics were analysed to reveal the respective indication area and treatment duration of cefiderocol, ceftazidime-avibactam, ceftolozane-tazobactam, and imipenem-cilastatin-relebactam. Acquisition costs were determined accurately on gram basis as they correlate with the drug amount and the treatment duration.

### 2.3. Statistical Analyses

Patient characteristics and reimbursement-relevant parameters were described by descriptive statistics. Bootstrapping of the overall (total) reimbursement was performed for 10,000 samples with a starting point for the Mersenne Twister at 1000. Statistical analyses were performed with Microsoft Excel (MS 365 Business Standard) and IBM SPSS Statistics software version 27 (IBM Corp., Armonk, NY, USA). Due to the 4-year period and for reasons of comparability, a discount rate of 3% to the year 2021 was applied [21]. Robustness of reimbursement data was tested through a structural sensitivity analysis, considering additional discount rates of 0%, 5%, and 10% [21]. All monetary values were given in Euro (EUR). The analysis was conducted based on the Consolidated Health Economic Evaluation Reporting Standards 2022 (CHEERS 2022) Statement (Table S1) [22].

## 3. Results

### 3.1. Drug-Associated Hospital Reimbursement Based on MDR-GNB

In total, 31 patients were identified through the retrospective reimbursement analysis of real-life data of the University Hospital Cologne. Clustered to MDR-GNB categories, *Enterobacterales* and *P. aeruginosa* built the numerically largest categories with 15 and 13 patients, respectively. One patient was exposed to *A. baumannii*. A mixed infection of *Enterobacterales* and *P. aeruginosa* was detected in two patients (Table 1). The median age of patients ranged from 58 to 66 years across categories while being 66 years overall. Almost two thirds (*n* = 19; 61.3%) of patients were male. Most patients were affiliated with statutory health insurances (*n* = 26; 83.9%). Both median length of stay (56 days) and median duration of mechanical ventilation (522 h) were longest in the *P. aeruginosa* category. A wide range was found in total treatment reimbursement (2566.52 EUR to 214,743.61 EUR) and case mix (0.74 points to 63.29 points) with median values across all categories of 40,243.24 EUR and 11.12 points, respectively.

Average drug-associated reimbursement of the most frequent G-DRG codes varied across MDR-GNB categories considering the cost category 4b (‘drugs—individual costs’) of the InEK cost matrix. For instance, G-DRG A36B was most frequently found in the *Enterobacterales* category (*n* = 4; 26.7%), accounting for average drug reimbursement of 1235.77 EUR. Drug-associated reimbursement of G-DRG A09B of the *P. aeruginosa* category (*n* = 6; 46.2%) amounted to 2265.27 EUR. The patient of the *A. baumannii* category was assigned to G-DRG code A13D (*n* = 1; 100.0%) with average G-DRG drug reimbursement of 632.34 EUR. G-DRG codes A09B (*n* = 1; 50.0%) and E79A (*n* = 1; 50.0%) were found in the category of mixed infection of *Enterobacterales* and *P. aeruginosa* with average drug reimbursement of 2265.27 EUR and 44.50 EUR, respectively (Tables S2–S5).

### 3.2. Determination of Last Resort Antibiotics Acquisition Costs

Cefiderocol, ceftazidime-avibactam, ceftolozane-tazobactam, and imipenem-cilastatin-relebactam are appropriate for treating *P. aeruginosa* and *Enterobacterales*. Cefiderocol is additionally approved for *A. baumannii* complex. Treatment durations were almost identical, reaching from a minimum of seven and eight days to a maximum of 14 days according to product information. Duration-specific acquisition costs of cefiderocol and imipenem-cilastatin-relebactam (minimum treatment duration: 7497.00 EUR and 7913.51 EUR; maximum treatment duration: 14,994.00 EUR and 15,827.01 EUR) were comparable. Differences were found for ceftazidime-avibactam and ceftolozane-tazobactam (minimum treatment duration: 3284.40 EUR and 5226.48 EUR; maximum treatment duration: 5747.70 EUR and 9146.34 EUR) (Table 2).

### 3.3. Quantification of Potential Funding Gap

**Figure 1:**
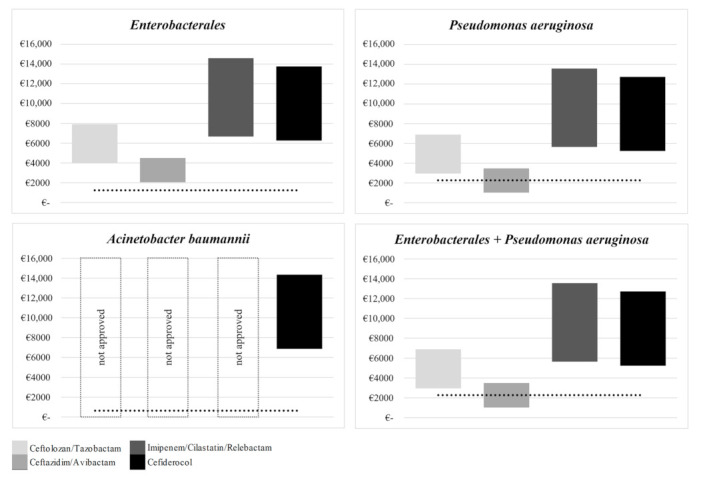
Drug-specific underfunding by MDR-GNB categories.

Underfunding was found in each individual MDR-GNB category when merging the results of both drug-associated hospital reimbursement and last resort antibiotics acquisition costs. For instance, the funding gap for treating *Enterobacterales* in G-DRG A36B reached from 2048.63 EUR in ceftazidime-avibactam at minimum treatment duration of eight days to 14,591.24 EUR in imipenem-cilastatin-relebactam in maximum treatment duration of 14 days. G-DRG A09B (2265.27 EUR) was most frequently found in two categories: *P. aeruginosa* and in mixed infection of *P. aeruginosa* and *Enterobacterales*. Both categories were underfunded, reaching from 1019.13 EUR in ceftazidime-avibactam to 13,561.74 EUR in imipenem-cilastatin-relebactam in minimum and maximum treatment duration, respectively. An underfunding of 6864.66–14,361.66 EUR was found for the treatment of *A. baumannii* including G-DRG A13D. Cefiderocol was considered an exclusive treatment option in this category based on EMA approval (Figure 1).

## 4. Discussion

This analysis examined the funding of last resort antibiotics in ICU patients with pneumonia due to MDR-GNB. Drug acquisition costs were compared with drug-associated reimbursement by G-DRG codes from a German hospital management perspective. A consistent underfunding of last resort antibiotics was identified, reaching from 1019.13 EUR (*P. aeruginosa*; mixed infection of *P. aeruginosa* and *Enterobacterales*) to 14,591.24 EUR (*Enterobacterales*). An appropriate antimicrobial therapy is essential to prevent non-response, side-effects, and resistance development [23]. The current guideline of the European Respiratory Society (ERS), European Society of Intensive Care Medicine (ESICM), European Society of Clinical Microbiology and Infectious Diseases (ESCMID), and Asociación Latinoamericana del Tórax (ALAT) recommends broad-spectrum empiric therapy against *P. aeruginosa* and extended-spectrum beta-lactamase (ESBL)-producing organisms of patients with HAP/VAP. The guideline panel recommends an initial combination therapy to cover Gram-negative bacteria, including methicillin-resistant *Staphylococcus aureus*, for high-risk HAP/VAP patients. The use of narrow-spectrum antibiotics is recommended for patients with suspected low risk of resistance and early-onset HAP/VAP [24].

In 2008, the German Ministry of Health developed a national antibiotic resistance strategy to counteract the scarcity of effective treatment options against MDR-GNB. An antibiotic stewardship program aiming at the rational and responsible use of antibiotics based on resistance testing was implemented to reduce an expansion of antimicrobial resistant pathogens [25]. The World Health Organisation has analysed antibacterial agents in clinical and preclinical development and concluded that too few new antibiotic compounds are expected [26]. The consistent underfunding of last resort antibiotic therapies through G-DRG tariffs, as shown in the current study, renders the prescription uneconomic for hospitals. Consequently, the research and development of new antimicrobial drugs by pharmaceutical companies is financially disincentivised. For many years, InEK consistently rejects extra-budgetary financing of antibiotic therapies as they are considered to be either sufficiently reimbursed or individual exceptions [27]. For antifungals though, hospitals receive extra-budgetary financing. Applications by medical scientists to implement a targeted reimbursement of last resort antibiotic therapies in accordance with the antibiotic stewardship principles have still not been approved [28]. As an alternative to last resort antibiotics, as extra-budgetary funding, the G-DRG system could break down G-DRG tariffs in more detail. From a hospital management perspective, this would prevent costly outliers and would thus counteract funding gaps.

Yet, inappropriate antimicrobial therapy may affect both medical and economic outcomes due to disease progression and prolonged hospitalisation [23,29]. In addition, the risk of readmission increases in patients with inappropriate therapy [30]. Comparable results were presented from various countries and settings [31,32].

### Methodological Considerations

Pathogen detection and antibiotic susceptibility testing may not be executed regularly or may lack clinical documentation due to insufficient reimbursement. It can therefore be assumed that the number of patients with MDR-GNB may be underestimated in this study. The G-DRG system clusters medically homogeneous cases with similar resource consumption by using reimbursement flat rates. Therefore, it cannot be ruled out that other acute comorbidities besides pneumonia might have influenced the overall treatment costs. Further, the administration of last resort antibiotics is not associated with a specific OPS code, which in turn may affect the G-DRG grouping. Thus, analysed cases showed a wide spectrum of different G-DRG codes and corresponding reimbursement. As underlying real-life data was retrieved tertiary care hospital, case mixes may be higher than in smaller hospitals due to higher severities of the cases. Drug-associated reimbursement was exemplarily evaluated for the most frequent G-DRG codes of each MDR-GNB category. Reimbursement may vary in other G-DRG codes which were not analysed. Yet, the consistent underfunding in all analysed G-DRG codes indicated a system-related issue. Acquisition costs of last resort antibiotics were calculated precisely but may be underestimated as the discarding of already opened but not fully used packaging remained unconsidered.

## 5. Conclusions

The current study demonstrates that the administration of last resort antibiotics is an underfunded measure in the treatment of ICU patients with MDR-GNB. The research and development of last resort antibiotics is driven by pharmaceutical companies and research hospitals, but lacking reimbursement hampers routine use in the clinic. Underfunding further accelerates the prescription of inappropriate antibiotics, and thus the ever-increasing number of multi-drug resistant bacteria. This analysis may thus contribute to the health–political debate as it identifies underfunding as a potential lever in breaking this cycle. Even stricter and globally mandatory antibiotic stewardship programs might help to implement clinically and economically sound strategies. Further analyses are needed to confirm the underfunding of last resort antibiotics in the ICU treatment of patients with MDR-GNB. These should include larger cohorts or consider potential differences across international reimbursement systems.

## Figures and Tables

**Table 1:** Baseline patient characteristics and hospital reimbursement-relevant treatment parameters.

	*Enterobacterales*	*P. aeruginosa*	*A. baumannii*	*Enterobacterales* and *P. aeruginosa* Mixed Infection	Total
Cases (Patients)	15 (15)	13 (13)	1 (1)	2 (2)	31 (31)
Age [in years]					
Median (range)	66 (28–78)	63 (40–76)	66	58 (44–72)	66 (28–78)
Mean (95% CI)	60.6 (53–68)	62.5 (57–68)	66	58	61.4 (57–66)
Gender					
Female (%)	4 (26.67)	7 (53.85)	-	1 (50)	12 (38.71)
Male (%)	11 (73.33)	6 (46.15)	1 (100)	1 (50)	19 (61.29)
Health Insurance [in n]					
Private (%)	3 (20.00)	2 (15.38)	-	-	5 (16.13)
Statutory (%)	12 (80.00)	11 (84.62)	1 (100)	2 (100)	26 (83.87)
Length of stay [in days]					
Median (range)	26 (8–106)	56 (4–146)	7	33.5 (7–60)	34 (4–146)
Mean (95% CI)	35.4 (21.9–48.9)	54.6 (31.5–77.7)	7	33.5	42.4 (30.0–54.8)
Duration of mechanical ventilation [in hours]					
Median (range)	136 (0–697)	522 (0–1023)	162	284 (7–561)	372 (0–1023)
Mean (95% CI)	261 (138.6–383.8)	509.6 (345.9–673.3)	162	284	360.4 (257.0–463.9)
Total treatment reimbursement [in EUR]					
Median (range)	33,833.61 (4048.55–147,114.86)	76,001.08 (3145.29–214,743.61)	39,468.50	40,984.08 (2566.52–79,401.64)	40,243.24 (2566.52–214,743.61)
Mean (95% CI)	43,595.64 (24,990.51–62,200.77)	84,852.25 (48,570.87–121,133.64)	39,468.50	40,984.08	60,595.18 (42,620.93–80,346.00)
Sensitivity analysis of total treatment reimbursement with different discount rates [in EUR]					
0 percent [median (range)]	37,348.66 (4556.68–156,074.16)	85,539.88 (3239.65–241,695.83)	40,652.55	43,562.92	43,974.87 (2888.64–214,695.83)
5 percent [median (range)]	33,189.16 (3748.79–141,563.86)	72,464.56 (3085.38–198,843.76)	38,716.71	39,391.06	38,716.71 (2376.49–198,843.76)
10 percent [median (range)]	31,680.56 (3112.27–128,986.91)	66,026.60 (2945.14–165,081.50)	36,956.86	35,795.25	34,280.38 (1972.98–165,081.50)
Case mix index [in points]					
Median (range)	7.51 (1.39–38.51)	21.88 (0.74–63.29)	6.09	12.34 (0.86–23.82)	11.12 (0.74–63.29)
Mean (95% CI)	11.00 (5.79–16.21)	21.64 (11.94–31.35)	6.09	12.34	15.40 (10.22–20.56)

**Table 2:** Last resort antibiotic acquisition costs by treatment duration.

Active Substance of Last Resort Antibiotic	Effective against [Inter Alia]	Minimum Treatment Duration [in Days] ^a^	Minimum Treatment Costs [in EUR] ^b^	Maximal Treatment Duration [in Days] ^a^	Maximal Treatment Costs [in EUR] ^b^
Cefiderocol	*Enterobacterales*; *P. aeruginosa*; *A. baumannii* complex	7	7497.00	14	14,994.00
Ceftazidime-Avibactam	*Enterobacterales*; *P. aeruginosa*	8	3284.40	14	5747.70
Ceftolozane-Tazobactam	*Enterobacterales*; *P. aeruginosa*	8	5226.48	14	9146.34
Imipenem-Cilastatin-Relebactam	*Enterobacterales* (except *Morganellaceae*); *P. aeruginosa*	7	7913.51	14	15,827.01

^a^ based on European Medicines Agency label of respective medical preparation, ^b^ based on German Lauer Taxe (reference list for pharmaceutical price information).

## Data Availability

Cost data of this analysis are included in this article in aggregated form. Patient-individual cost data will not be made available to others.

## Supplementary Materials

The following supporting information can be downloaded at: https://www.mdpi.com/article/10.3390/healthcare10122546/s1, Table S1: CHEERS 2022 Checklist, Table S2: InEK cost matrix of G-DRG code A09B based on G-DRG-Report-Browser 2021 in Euro, Table S3: InEK cost matrix G-DRG code A13D based on G-DRG-Report-Browser 2021 in Euro, Table S4; InEK cost matrix G-DRG code A36B based on G-DRG-Report-Browser 2021 in Euro, Table S5: InEK cost matrix G-DRG code E79A based on G-DRG-Report-Browser 2021 in Euro.

## Abbreviations

Abbreviation	Definition
DRG	Diagnosis-related groups
G-DRG	German diagnosis-related groups
HAP	Hospital-acquired pneumonia
ICU	Intensive care unit
InEK	German Institute for Remuneration in Hospitals
MDR-GNB	Multidrug-resistant Gram-negative bacteria
OPS	Operation and procedure
RKI	Robert Koch-Institute
VAP	Ventilator-associated pneumonia
vHAP	Hospital-acquired pneumonia requiring mechanical ventilation
