# Supplementary material for: Last Resort Antibiotics Costs and Reimbursement Analysis of Real-Life ICU Patients with Pneumonia Caused by Multidrug-Resistant Gram-Negative Bacteria in Germany
Source: Healthcare (Basel). 2022 Dec 15;10(12):2546. doi: 10.3390/healthcare10122546 (PMC9778355; doi:10.3390/healthcare10122546)
Supplement: Supplementary file 1 [file healthcare-10-02546-s001.zip › healthcare-2074141-supplementary.pdf]

Healthcare

Last resort antibiotics costs and reimbursement analysis of real-life ICU patients with pneumonia caused by multidrug-resistant gram-negative bacteria in Germany

Julia Jeck[1,2], Sebastian M. Wingen-Heimann[2,3], Florian Jakobs[4], Jennifer Franz[1,2,5], Christoph T. Baltin[1,3,6], Anna Kron[1,2,5,7], Boris Böll[2], Matthias Kochanek[2,5], Oliver A. Cornely[2,5,8,9,10], Florian Kron[1,2,3,5]

Affiliations

[1] VITIS Healthcare Group, Cologne, Germany

[2] University of Cologne, Faculty of Medicine and University Hospital Cologne, Department I of Internal Medicine, Cologne, Germany

[3] FOM University of Applied Sciences, Essen, Germany

[4] University of Duisburg-Essen, Faculty of Medicine and Essen University Hospital, Department of Haematology and Stem Cell Transplantation, Essen, Germany

[5] University of Cologne, Faculty of Medicine and University Hospital Cologne, Center for Integrated Oncology (CIO ABCD), Cologne, Germany

[6] University of Cologne, Faculty of Medicine and University Hospital Cologne, Department of Orthopedics and Trauma Surgery, Cologne, Germany

[7] National Network Genomic Medicine Lung Cancer, University Hospital Cologne, Cologne, Germany

[8] University of Cologne, Faculty of Medicine and University Hospital Cologne, Clinical Trials Centre Cologne (ZKS Köln), Cologne, Germany

[9] University of Cologne, Faculty of Medicine and University Hospital Cologne, Translational Research, Cologne Excellence Cluster on Cellular Stress Responses in Aging-Associated Diseases (CECAD), Cologne, Germany

[10] University of Cologne, Faculty of Medicine and University Hospital Cologne, Excellence Center for Medical Mycology (ECMM), Cologne, Germany

Corresponding author

Prof. Dr. Florian Kron
FOM University of Applied Sciences
Herkulesstraße 32
45127 Essen, Germany
Phone: +49 176 6200 3950
Email: florian.kron@fom.de

Table S1 CHEERS 2022 Checklist

Topic	No.	Item	Location where item is reported
**Title**	1	Identify the study as an economic evaluation and specify the interventions being compared.	page 1
**Abstract**	2	Provide a structured summary that highlights context, key methods, results, and alternative analyses.	page 2
**Introduction**			
Background and objectives	3	Give the context for the study, the study question, and its practical relevance for decision making in policy or practice.	page 2-3
**Methods**			
Health economic analysis plan	4	Indicate whether a health economic analysis plan was developed and where available.	n/a
Study population	5	Describe characteristics of the study population (such as age range, demographics, socioeconomic, or clinical characteristics).	page 3
Setting and location	6	Provide relevant contextual information that may influence findings.	page 3
Comparators	7	Describe the interventions or strategies being compared and why chosen.	page 3
Perspective	8	State the perspective(s) adopted by the study and why chosen.	page 3
Time horizon	9	State the time horizon for the study and why appropriate.	page 3
Discount rate	10	Report the discount rate(s) and reason chosen.	page 4
Selection of outcomes	11	Describe what outcomes were used as the measure(s) of benefit(s) and harm(s).	n/a
Measurement of outcomes	12	Describe how outcomes used to capture benefit(s) and harm(s) were measured.	n/a
Valuation of outcomes	13	Describe the population and methods used to measure and value outcomes.	n/a
Measurement and valuation of resources and costs	14	Describe how costs were valued.	page 3
Currency, price date, and conversion	15	Report the dates of the estimated resource quantities and unit costs, plus the currency and year of conversion.	pages 3-4
Rationale and description of model	16	If modelling is used, describe in detail and why used. Report if the model is publicly available and where it can be accessed.	n/a
Analytics and assumptions	17	Describe any methods for analysing or statistically transforming data, any extrapolation methods, and approaches for validating any model used.	page 4
Characterising heterogeneity	18	Describe any methods used for estimating how the results of the study vary for subgroups.	n/a
Characterising distributional effects	19	Describe how impacts are distributed across different individuals or adjustments made to reflect priority populations.	n/a
Characterising uncertainty	20	Describe methods to characterise any sources of uncertainty in the analysis.	page 4
Approach to engagement with patients and others affected by the study	21	Describe any approaches to engage patients or service recipients, the general public, communities, or stakeholders (such as clinicians or payers) in the design of the study.	n/a
**Results**			
Study parameters	22	Report all analytic inputs (such as values, ranges, references) including uncertainty or distributional assumptions.	pages 5 and 7
Summary of main results	23	Report the mean values for the main categories of costs and outcomes of interest and summarise them in the most appropriate overall measure.	pages 6-7
Effect of uncertainty	24	Describe how uncertainty about analytic judgments, inputs, or projections affect findings. Report the effect of choice of discount rate and time horizon, if applicable.	Table 1
Effect of engagement with patients and others affected by the study	25	Report on any difference patient/service recipient, general public, community, or stakeholder involvement made to the approach or findings of the study	n/a
**Discussion**			
Study findings, limitations, generalisability, and current knowledge	26	Report key findings, limitations, ethical or equity considerations not captured, and how these could affect patients, policy, or practice.	pages 7-8
**Other relevant information**			
Source of funding	27	Describe how the study was funded and any role of the funder in the identification, design, conduct, and reporting of the analysis	page 9
Conflicts of interest	28	Report authors conflicts of interest according to journal or International Committee of Medical Journal Editors requirements.	page 9

Table S2 InEK cost matrix of G-DRG code A09B based on G-DRG-Report-Browser 2021 in Euro

	Labour Costs			Material Costs						Labour / Material Costs		
	Physicians	Nursing	Medical / technical staff	Drugs		Implants	Further material demand			Medical infrastructure	Non-medical infrastructure	
				General	Individual		General	Individual	Third parties			
Costs per case	1	2	3	4a	4b	5	6a	6b	6c	7	8	Total
Ward	365.01	0.00	24.91	43.83	82.99	0.00	48.13	11.65	11.05	142.40	346.01	975.98
Intensive care unit	12,082.18	0.00	378.65	2,118.42	1,332.88	4.07	4,702.58	200.76	111.34	3,353.16	8,352.48	32,636.52
Operating rooms	1,219.15	0.00	932.27	53.64	81.37	915.77	553.45	469.31	76.82	562.73	752.01	5,616.52
Anaesthesia	870.29	0.00	546.01	51.90	17.12	0.00	189.29	7.27	2.22	114.93	264.90	2,063.93
Cardiac diagnostics/ therapy	65.16	0.00	61.25	2.97	0.45	274.95	39.61	67.06	10.69	32.06	52.16	606.36
Endoscopic diagnostics/ therapy	120.96	0.00	132.28	4.54	0.79	3.10	53.72	45.92	1.84	72.27	90.50	525.92
Radiology	506.40	0.00	472.27	9.21	4.14	89.07	91.02	24..93	65.53	215.30	341.58	2,037.45
Laboratories	209.42	0.00	840.50	17.73	726.60	15.72	705.61	24.94	810.32	118.12	377.43	3,846.39
Further diagnostics	137.96	1.36	95.04	2.41	0.00	0.00	12.55	0.79	9.70	25.81	54.33	339.95
Therapeutic procedures	33.25	14.34	689.77	0.80	0.00	0.00	5.86	2.72	163.26	26.64	195.65	1,132.28
Patient admission	61.17	3.54	43.75	2.10	18.93	0.00	6.64	0.02	0.17	12.86	38.40	187.58
Total	15,570.94	19.24	4,216.70	2.,307.55	2,265.27	1,302.68	6,408.46	1,075.37	1,260.94	4,676.28	10,865.45	49,968.88

Abbreviations: G-DRG, German diagnosis-related groups; InEK, German Institute for the Hospital Remuneration System

Definition: Ventilation >499 hours or >249 hours with complex intensive care treatment > 2352/1932/2208 points, with congenital malformation or tumour disease, age <3 years or with highly complex procedure or with complex operating room procedure or complex intensive care treatment > 1764/1932 / - points, age <16 years

Table S3 InEK cost matrix G-DRG code A13D based on G-DRG-Report-Browser 2021 in Euro

	Labour Costs			Material Costs						Labour / Material Costs		
	Physicians	Nursing	Medical / technical staff	Drugs		Implants	Further material demand			Medical infrastructure	Non-medical infrastructure	
				General	Individual		General	Individual	Third parties			
Costs per case	1	2	3	4a	4b	5	6a	6b	6c	7	8	Total
Ward	491.11	0.00	62.50	62.27	56.44	0.00	67.92	9.02	28.10	272.73	690.70	1,740.79
Intensive care unit	3,357.93	0.00	82.14	544.56	293.39	0.04	1,170.15	54.75	25.68	972.22	2,478.53	8,979.39
Operating rooms	830.92	0.00	691.42	31.97	30.92	504.70	380.70	393.52	11.96	360.85	485.75	3,722.71
Anaesthesia	585.14	0.00	378.90	34.75	5.03	0.00	118.20	2.84	0.11	77.61	183.88	1,386.46
Cardiac diagnostics/ therapy	82.66	0.00	92.79	4.54	0.16	248.42	36.99	142.79	12.86	42.73	67.43	731.37
Endoscopic diagnostics/ therapy	37.09	0.00	46.07	1.45	0.06	3.25	19.26	8.46	0.20	22.03	29.38	167.25
Radiology	287.21	0.00	259.49	4.29	3.07	30.18	53.88	228.30	47.90	115.78	180.39	1,240.49
Laboratories	75.25	0.00	286.54	6.70	236.12	0.00	227.72	4.02	312.98	41.16	133.16	1.324.65
Further diagnostics	68.06	1.03	49.72	0.87	0.01	0.00	5.75	0.43	2.80	11.40	28.48	168.55
Therapeutic procedures	22.86	7.47	206.11	0.64	0.00	0.00	2.86	0.61	58.98	10.19	61.84	371.56
Patient admission	55.83	5.88	39.35	2.08	7.14	0.00	6.45	0.51	0.56	11.27	36.29	165.36
Total	5,895.06	14.38	2,195.03	694.12	632.34	816.59	2,089.88	845.25	502.13	1,937.97	4,375.83	19,998.58

Abbreviations: G-DRG, German diagnosis-related groups; InEK, German Institute for the Hospital Remuneration System

Definition: Ventilation >95 hours with complex operating room procedure, age >5 years or with complex intensive care treatment > - / 828 / - points or complex operating room procedure or with certain operating room procedure or complicating constellation or with complex intensive care treatment >588/552/552 points or age <16 years with malignant neoplasm

Table S4 InEK cost matrix G-DRG code A36B based on G-DRG-Report-Browser 2021 in Euro

	Labour Costs			Material Costs						Labour / Material Costs		
	Physicians	Nursing	Medical / technical staff	Drugs		Implants	Further material demand			Medical infrastructure	Non-medical infrastructure	
				General	Individual		General	Individual	Third parties			
Costs per case	1	2	3	4a	4b	5	6a	6b	6c	7	8	Total
Ward	670.13	0.00	70.50	183.52	257.44	0.00	137.71	23.55	38.21	395.22	930.79	2,707.07
Intensive care unit	4,869.39	0.00	191.30	824.11	551.30	0.02	1,739.39	62.69	30.91	1,553.87	3,815.69	13,638.67
Operating rooms	424.75	0.00	362.95	12.80	11.80	41.90	152.41	107.09	0.67	194.22	267.34	1,576.01
Anaesthesia	336.82	0.00	215.82	19.35	1.50	0.00	65.61	1.56	0.31	46.50	105.24	792.71
Delivery ward	0.12	0.00	0.39	0.03	0.00	0.00	0.04	0.00	0.00	0.08	0.30	0.96
Cardiac diagnostics/ therapy	11.26	0.00	10.23	0.39	0.06	1.72	4.58	19.93	0.95	4.07	7.71	60.90
Endoscopic diagnostics/ therapy	59.42	0.00	65.42	2.48	0.32	0.84	26.07	14.51	0.00	33.47	46.19	248.72
Radiology	214.77	0.00	197.67	2.83	0.65	9.69	31.89	39.90	46.67	86.96	135.97	767.00
Laboratories	161.69	0.00	541.66	8.97	473.02	27.85	448.44	5.78	530.93	74.30	248.06	2,520.70
Further diagnostics	86.47	1.22	56.52	1.11	0.09	0.00	8.10	0.53	6.07	16.27	35.41	211.79
Therapeutic procedures	21.22	15.04	317.53	0.84	0.00	0.00	2.82	2.63	98.04	15.46	94.79	568.37
Patient admission	57.86	3.53	38.17	2.34	3.10	0.00	6.62	0.07	0.18	13.58	37.16	162.61
Total	6,913.90	19.79	2,068.16	1,058.77	1,299.36	82.02	2,623.68	278.24	752.94	2,434.00	5,724.65	23,255.51

Abbreviations: G-DRG, German diagnosis-related groups; InEK, German Institute for the Hospital Remuneration System

Definition: Complex intensive care treatment >588/552/828 and <981/1105/1657 points for certain diseases and disorders or complicating constellations in the case of failure and rejection of a transplant of hematopoietic cells

Table S5 InEK cost matrix G-DRG code E79A based on G-DRG-Report-Browser 2021 in Euro

	Labour Costs			Material Costs						Labour / Material Costs		
	Physicians	Nursing	Medical / technical staff	Drugs		Implants	Further material demand			Medical infrastructure	Non-medical infrastructure	
				General	Individual		General	Individual	Third parties			
Costs per case	1	2	3	4a	4b	5	6a	6b	6c	7	8	Total
Ward	585.26	0.00	38.86	103.05	18.58	0.00	89.19	2.53	6.79	306.59	848.63	1,999.48
Intensive care unit	157.83	0.00	2.80	24.58	4.13	0.00	44.52	0.33	0.48	52.04	141.95	428.66
Operating rooms	1.94	0.00	1.87	0.07	0.01	0.19	0.80	0.66	0.17	1.20	1.42	8.33
Anaesthesia	2.62	0.00	1.49	0.12	0.00	0.00	0.42	0.01	0.03	0.32	0.67	5.68
Cardiac diagnostics / therapy	1.95	0.00	2.14	0.10	0.00	0.01	0.87	1.78	0.03	0.85	1.42	9.15
Endoscopic diagnostics/ therapy	29.92	0.00	34.34	1.23	0.10	0.33	12.97	4.42	0.12	14.70	19.81	117.94
Radiology	50.27	0.00	50.85	0.70	0.12	0.03	6.95	2.42	28.29	19.17	31.58	190.38
Laboratories	18.41	0.00	80.18	1.76	21.39	0.00	62.84	0.24	78.36	9.94	34.41	307.53
Further diagnostics	44.49	1.11	31.69	0.76	0.06	0.00	5.13	0.23	0.81	8.27	19.30	111.85
Therapeutic procedures	4.90	4.04	80.49	0.13	0.00	0.00	0.98	0.34	7.89	3.12	25.72	127.61
Patient admission	57.00	6.33	37.91	2.05	0.11	0.00	7.04	0.03	0.27	10.86	36.85	158.45
Total	954.59	11.48	362.62	134.55	44.50	0.56	231.71	12.99	123.24	427.06	1,161.76	3,465.06

Abbreviations: G-DRG, German diagnosis-related groups; InEK, German Institute for the Hospital Remuneration System

Definition: Infections and inflammations of the respiratory organs with a complex diagnosis or extremely severe complications or comorbidities, length of stay > 1 day or with extremely severe complications or comorbidities with certain infections or inflammations
